# Amino Acid Signature in Human Melanoma Cell Lines from Different Disease Stages

**DOI:** 10.1038/s41598-018-24709-0

**Published:** 2018-04-19

**Authors:** Christine Wasinger, Alexandra Hofer, Oliver Spadiut, Martin Hohenegger

**Affiliations:** 1 Institute of Pharmacology, Center for Physiology and Pharmacology, Medical University Vienna, Waehringerstrasse 13A, A-1090 Vienna, Austria; 2 Institute for Chemical, Enviromental and Biological Engineering, TU Wien, Gumpendorferstrasse 1a, A-1060 Wien, Austria

## Abstract

Cancer cells rewire metabolism to sustain high proliferation rates. Beside glycolysis and glutaminolysis, amino acids substitute as energy source, feed fatty acid biosynthesis and represent part of the secretome of transformed cells, including melanoma. We have therefore investigated acetate, pyruvate and the amino acid composition of the secretome of human melanoma cells representing the early slow (WM35, WM278, WM793b and VM21) and metastatic fast (A375, 518a2, 6F and WM8) growth phase in order to identify possible signalling components within these profiles. Proliferation assays and a principle component analysis revealed a stringent difference between the fast and slow growing melanoma cells. Moreover, upon inhibition of the mevalonate pathway, glutamic acid and alanine were identified as the central difference in the conditional media. A supplementation of the media with glutamic acid and the combination with alanine significantly accelerated the proliferation, migration and invasion of early stage melanoma cells, but not metastatic cells. Finally, the inhibition of the mevalonate pathway abolished the growth advantage of the melanoma cells in a time dependent manner. Taken together, these data corroborate a stage specific response in growth and aggressiveness to extracellular glutamic acid and alanine, indicative for microenvironmental signalling of individual amino acids.

## Introduction

Tumour signalling and progression is strongly dependent on the tumour microenvironment which comprises components like the extracellular matrix, surrounding stromal cells and signalling molecules including secreted proteins^1^. In melanoma immune checkpoint inhibitors were evaluated for the first time to highlight the microenvironment as a therapeutic battlefield for the immune system to attack transformed cells^2^. Moreover, metabolic reprogramming in response to oncogenic stimuli has been elucidated as an adaption mechanism to scope with hypoxia, acidosis and cellular stress in the tumour microenvironment^3,4^. Decoupling of the mitochondrial tricarboxylic acid (TCA) cycle from cytosolic glycolysis allows cancer cells to establish a flexible adaptation to the conditions of the microenvironment by glycolysis and glutaminolysis^5,6^.

On the crossroads of glycolysis and glutaminolysis, acetyl-CoA has been established to play a crucial role in cancer cell progression by feeding fatty acid synthesis and the mevalonate pathway^7^. The activation of the mevalonate pathway is therefore essential for a rapid proliferation of transformed cells and inhibition associated with cell cycle arrest and the induction of apoptosis^8–12^. Conversely, an activation of the mevalonate pathway is triggered by mutant p53 or Myc and thereby favours the conjecture that pharmacological inhibition by statins may serve as a therapeutic concept^7,12,13^. This assumption is further supported by the finding that the dysregulation of the mevalonate pathway promotes transformation^14^.

Using statins is a proper tool to trigger the mitochondrial pathway of apoptosis in various cancer cells^9,10,15^. Interestingly, human metastatic melanoma cells are highly susceptible to statin induced apoptosis, while cells from the radial growth phase and primary human melanocytes are virtually insensitive^8,16^. It is therefore anticipated that fast proliferation rates are in favour of mevalonate pathway inhibition and thereby may use a switch from glucose utilisation to glutamine^7^. Recently, amino acids other than glutamine were responsible for the majority of proliferative cell mass^17^. Amino acids substitute as energy source, feed lipid biosynthesis and represent part of the secretome of transformed cells, including melanoma. However, little is known whether extracellular amino acid profiles correlate with specific growth behaviour of defined melanoma cell lines. Melanoma are heterogeneous tumours with different subpopulations characterized by distinct doubling times^18^. We have therefore investigated the amino acid composition as well as acetate and pyruvate of the secretome of human melanoma cells representing early slow growth phase and rapid growth phase of metastatic cells. Making use of subsequent multivariate data analysis, namely principle component analysis (PCA) and partial least squares (PLS) regression enabled to elucidate significant changes in the amino acid composition of media in a time and stage dependent manner. Further analyses of proliferation, migration and invasion confirmed a crucial role for glutamic acid to support enhanced cell growth and aggressiveness in early stage melanoma cells. Inhibition of the mevalonate pathway abrogated the growth advantage and thereby underlined the importance of the mevalonate pathway in melanoma progression. Finally, the underlying mechanisms and potential therapeutic implications of our findings were discussed.

## Results

### Deviation in amino acid profiles characterize melanoma cells of different stages

**Figure 1:**
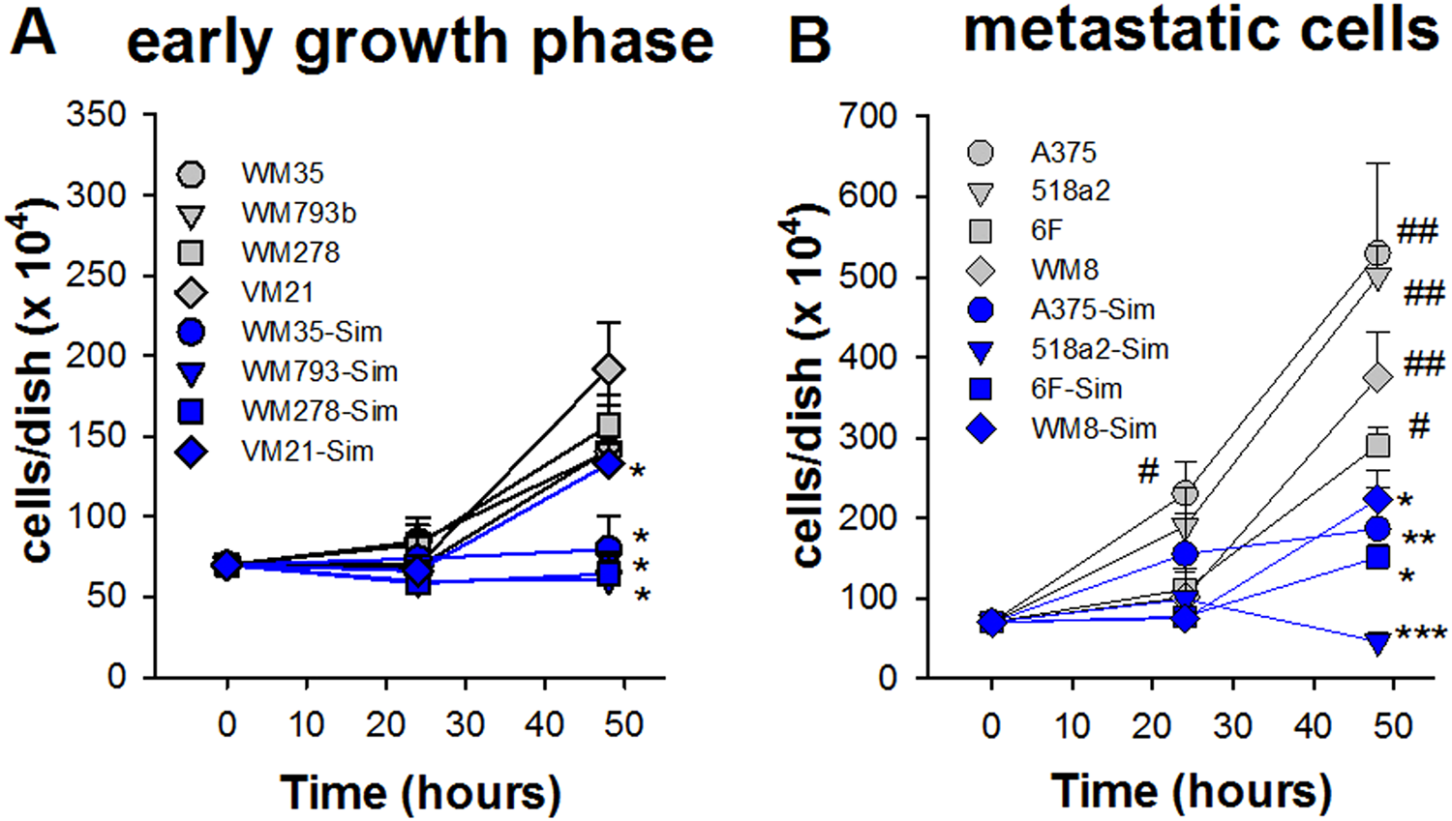
Simvastatin inhibits proliferation of human melanoma cells (WM35, WM278, WM793b, VM21, A375, 518a2, 6F and WM8). Slow (**A**) and fast (**B**) growing human melanoma cells were incubated in the absence and presence of 10 µM Simvastatin (Sim). At the indicated time points significant increase in proliferation under control conditions was analysed by ANOVA and post hoc Dunnett’s test (^#^p < 0.05; ^##^p < 0.01). Asterisks indicate statistical significance of Simvastatin treatment versus corresponding controls using Mann-Whitney Rank Sum Test (*p < 0.05; **p < 0.01).

Human metastatic melanoma cells (Fig. 1B) grow significantly faster than WM35, WM278, WM793b and VM21 cells from the early radial and vertical growth phase of primary tumours, i.e. within 48 hours proliferation was not significantly enhanced in slow growing cells (Fig. 1A). This biological criterion was used throughout this manuscript to distinguish between the two growth types of melanoma cells. Expression patterns of transcription factors like microphthalmia-associated transcription factor (MITF) and inversely correlated receptor tyrosine kinases like AXL have been implicated in staging of melanoma with respect to progression and resistance^19^. However, the expression levels of MITF in various melanoma cell lines are highly variable and correlation to other receptor tyrosine kinases may be also implicated in acquired drug resistance^20^. WM793b cells were selected from primary melanoma which lack metastatic potential in a SCID murine xenograft model^21^. Accordingly, based on the proliferation velocity depicted in Fig. 1, WM35, WM278, WM793b and VM21 cells were classified as slow growing melanoma cells while the others (A375, 518a2, WM8 and 6F cells) were termed fast growing and from metastatic origin. Importantly, inhibition of the mevalonate pathway by Simvastatin significantly reduced proliferation in all cell lines (Fig. 1). Next, we asked the question, whether it is possible to discriminate melanoma cell lines with different proliferation behaviour on the basis of extracellular amino acid profiles? The method of choice is principle component analysis (PCA), which enables to identify the principle components (e.g. individual amino acids or patterns of amino acids) out of a complex data set to explain biological differences between cells or treatments.

**Figure 2:**
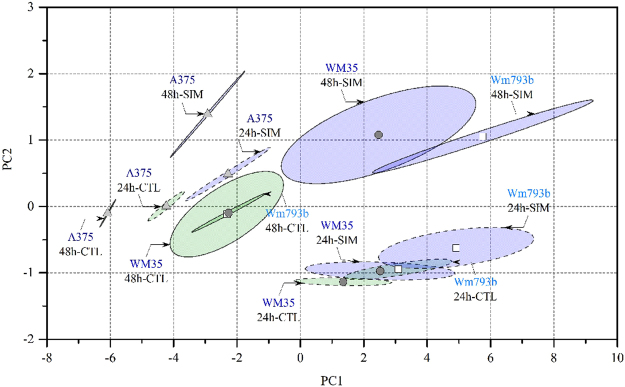
Overview of WM35, WM793b and A375 melanoma cells separated by incubation time and Simvastatin treatment. The amino acid concentrations listed in Supplementary Table S1 were used to generate PCA. WM35, WM793b and A375 melanoma cells were cultured in the absence (green) and presence (violet) of 10 µM Simvastatin for 24 h and 48 h. The model resulted in 5 PCs explaining 93.5% of the total variance by the first component (R^2^ cum of 0.997). In the simplified score plot the dots represent the center of each cluster and the ellipses represent the variance inside each cluster. The clusters of A375 cells are clearly separated from WM35 and WM793b cells. In the presence of Simvastatin treatment marked shifts toward PC2 are observed, indicating effects by components of the amino acid profiles.

Therefore, the conditional media of WM35, WM793b and A375 melanoma cells were used for HPLC analysis to determine acetate, pyruvate and amino acid profiles (Supplementary Table S1). The cells were treated in the absence and presence of Simvastatin for 24 and 48 hours. After normalization of the data to the cell count, principle component analysis (PCA) was performed in order to assess differences in the amino acid profiles according to cell line, incubation time and treatment via dimensionality reduction. As the difference between cell lines in acetate and pyruvate secretion is quite obvious due to different proliferation velocity, only amino acids were considered for pattern recognition in order to prevent overlaying effects by the organic acids. In the case of identical profiles for all cells and treatments the data sets would cluster in the centre of the plot around the coordinate origin. Clearly, this is not the case and a marked clustering was observed for A375 cells versus slowly growing cell lines WM35 and WM793b (Fig. 2). Stage dependent clustering of the cell lines was still retained after inhibition of the mevalonate pathway with Simvastatin. Moreover, clustering was also dependent on incubation time, indicating that metabolic rates determined for amino acids may provide further insights.

**Figure 3:**
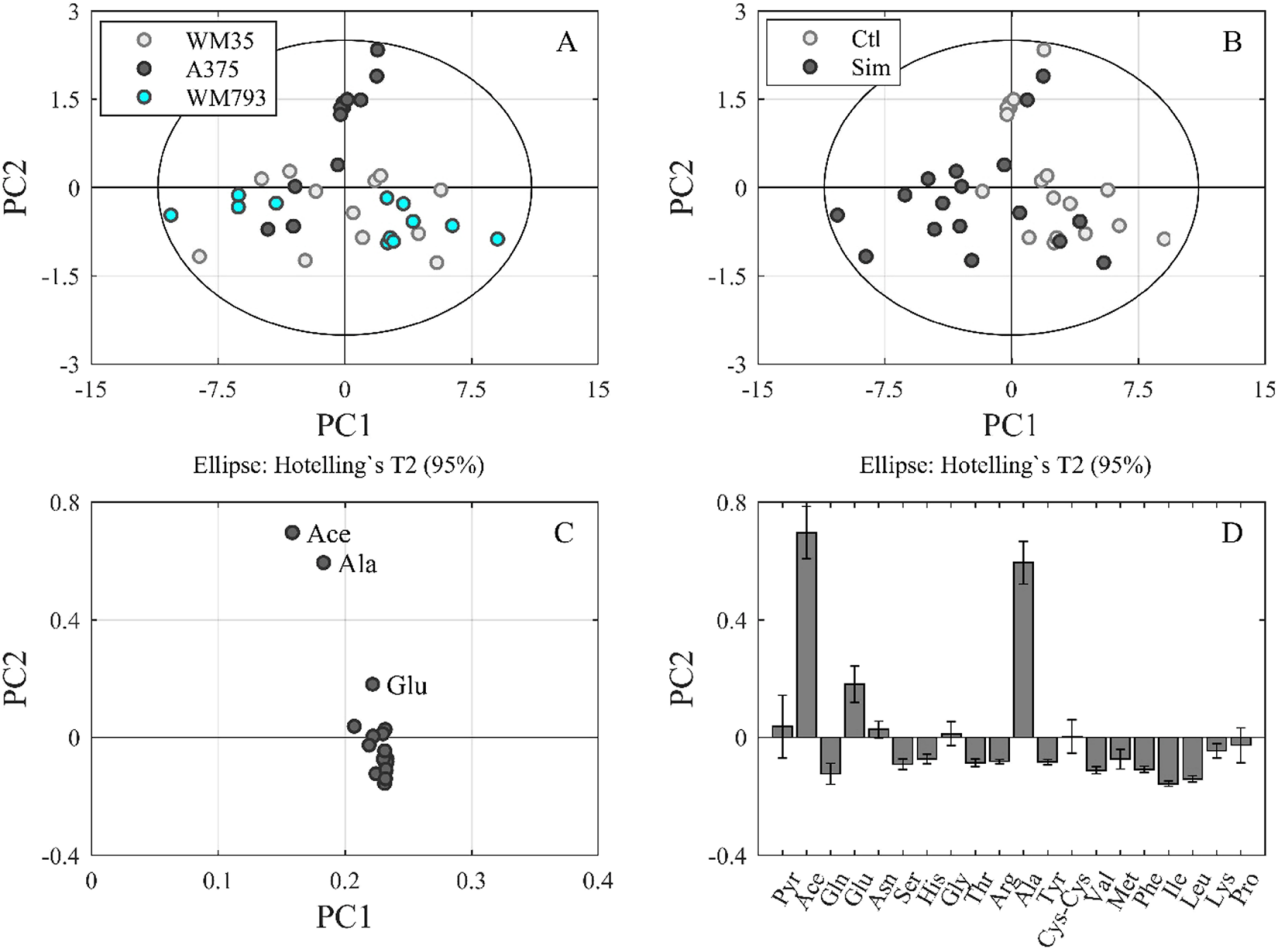
Differences between Simvastatin treated and untreated cells with respect to the specific rates of the metabolites. PCA of the specific metabolic rates between 24 h and 48 h of incubation were calculated for WM35, WM793b and A375 cells in the absence (Ctl) and presence of 10 µM Simvastatin. Four PCs were generated (R^2^ cum of 0.984 and Q^2^ cum of 0.943). The score plot of PC1 and PC2 is illustrated according to the individual cell lines (**A**) and according to Simvastatin treatment (**B**). The combination of the two score plots shows first the separation of A375 cells from the other cell lines along PC2, and second that this separation occurs especially for untreated cells (in greater detail treated and untreated cell lines are depicted in Supplementary Fig. S1). The loading plot (**C**) shows the variable distribution between PC1 and PC2, indicating that acetate, alanine and glutamic acid are the main contributors to the metabolic differences in the metastatic compared to the other cell lines. The loading plot (**D**) for PC2 again indicates the significance of the loading variables acetate, alanine and glutamic acid (calculated by the jack-knife algorithm).

The difference in amino acid concentrations of conditional media between the 24 and 48 hour values was used to determine metabolic rates of individual amino acids in order to improve comparability of the experimental results. PCA was performed after calculation of the specific rates, i.e. the change in amino acid (and organic acid) concentration per cell and hour, indicating uptake or secretion. The data dimension could be reduced to four principle components (PCs) explaining in total 98.4% of the variance in the data. PC1 mainly represents the differences between Simvastatin treated and untreated cells of the slow growing cells, whereas PC2 shows the differences of A375 cells from the WM35 and WM793b cells, respectively (Fig. 3A,B; Supplementary Fig. S1). The metabolic correlation for this clustering can be seen in the loading plots (Fig. 3C,D). Mainly, metastatic A375 cells seem to take up more acetate, alanine and glutamic acid than slow growing cells, because the PC2 values for A375 cells are highest (Fig. 3A).

**Figure 4:**
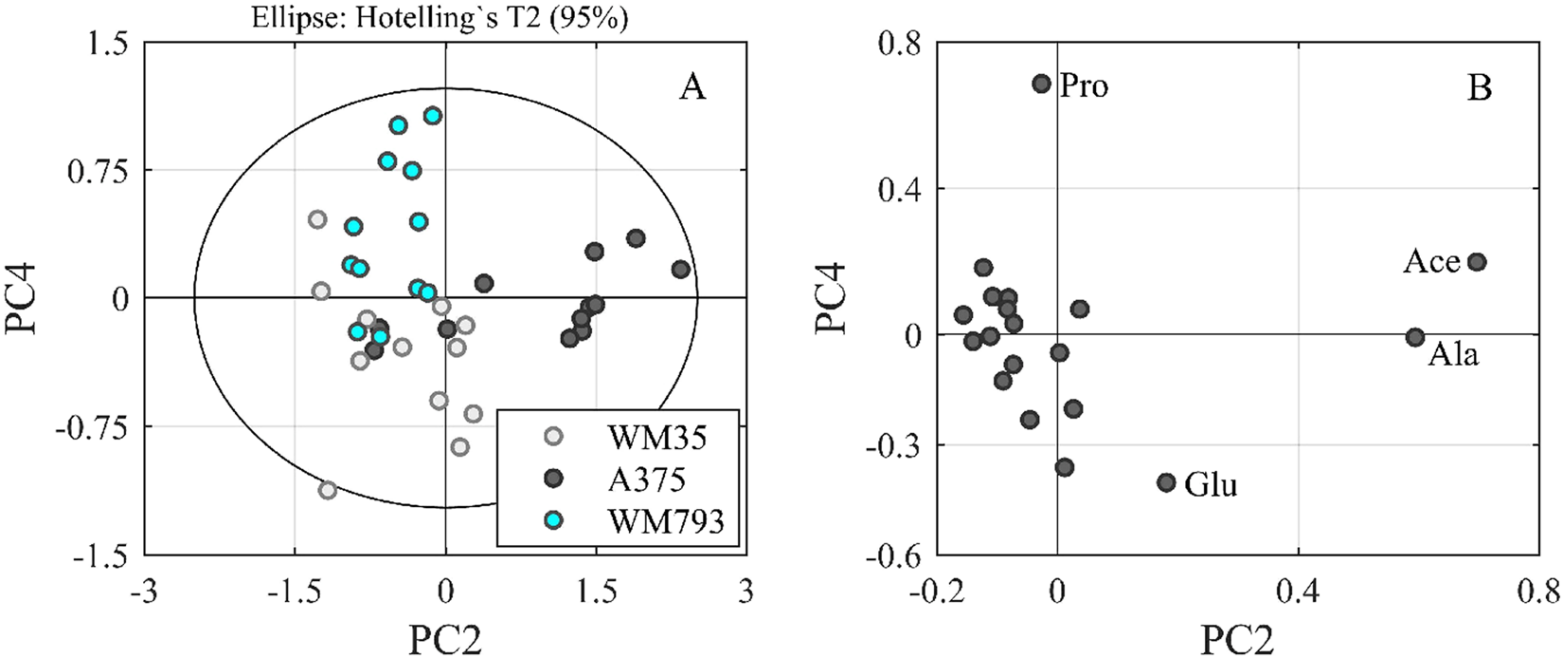
Uptake of proline by WM793b cells. PCA of the specific metabolic rates determined from 24 h and 48 h measurements results in four PCs (R^2^ cum of 0.984 and Q^2^ cum of 0.943). Score plot (**A**) shows the distribution of the cell lines (WM35, WM793b and A375) between PC2 and PC4. The loading plot (**B**) identifies proline (Pro) as metabolite that is especially taken up by WM793b cells as it shows a high loading on PC4, which corresponds to these cells in plot (**A**). Similarly, along PC2 metabolic differences of acetate (Ace) and alanine (Ala) may be attributed to the metastatic cell line A375.

Another interesting finding of the metabolite analysis in conditional media is obtained from WM793b cells and can also be extracted from the PCA model shown in Fig. 3. Each PC of the model describes a different variance in the dataset. Analysing the score plot of PC2 versus PC4 shows again the differences of A375 cells along PC2. Additionally, a separation of the WM35 and WM793b cells along PC4 can be observed (Fig. 4A). According to the loading plot (Fig. 4B) this separation correlates with proline, i.e. proline seems to be taken up in higher amounts by WM793b cells and to a lesser extent by WM35 cells. This finding may correlate to the observation by others that proline *de novo* synthesis is an important downstream feature of glutaminolysis in melanoma cells compared to melanocytes and contributes to redox homeostasis^5,22,23^.

### Impact of mevalonate pathway inhibition

**Figure 5:**
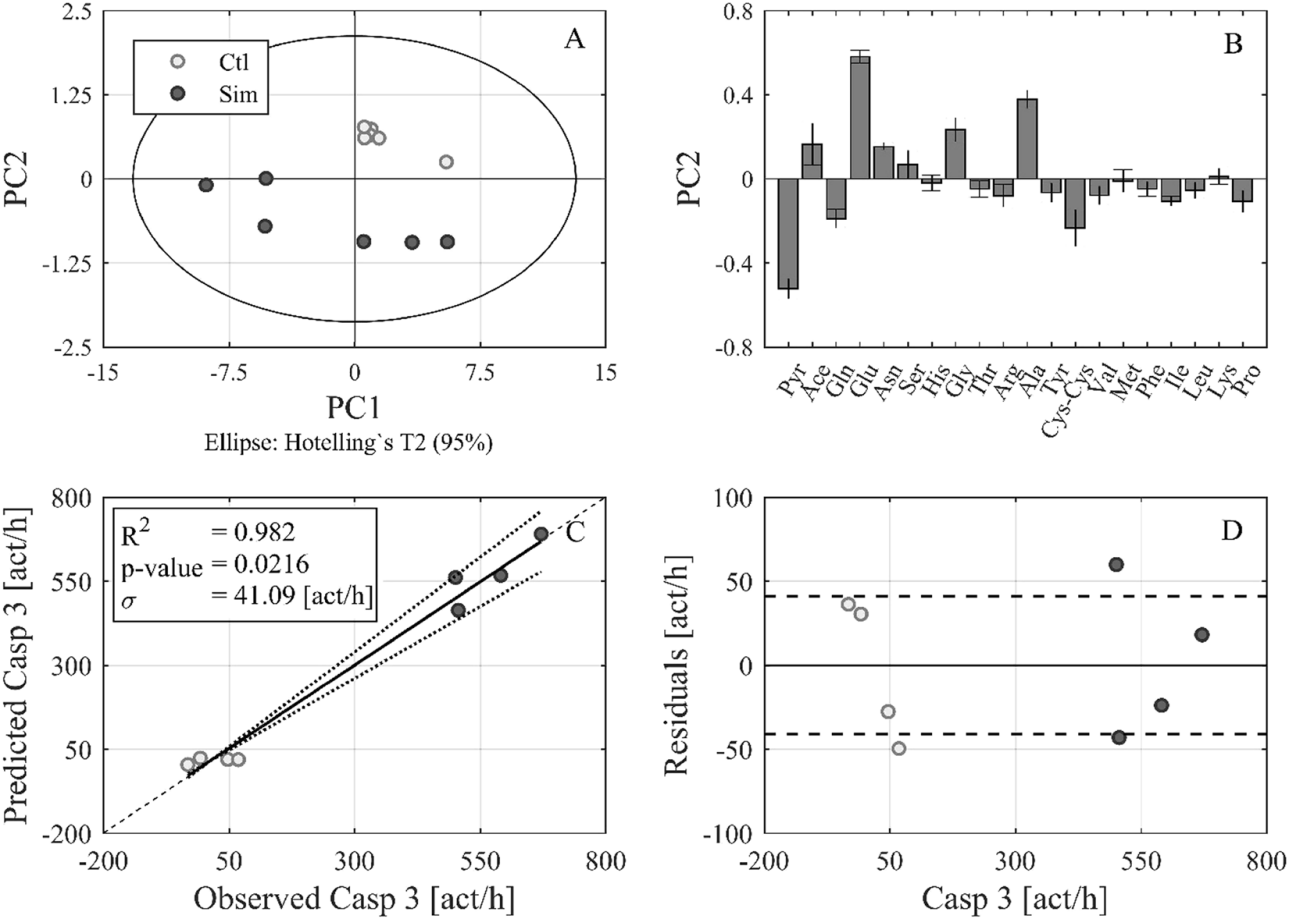
Impact of Simvastatin treatment on metabolism and caspase 3 activity in A375 cells. Plot (**A**,**B**) represent the score and loading plot of a PCA of the specific metabolic rates of Simvastatin treated (Sim) and untreated (Ctl) A375 cells. The dimension reduction resulted in 2 PCs (R^2^ cum of 0.993 and Q^2^ cum of 0.986). Clustering according to treatment along PC2 is clearly visible and can be assigned to a higher loading of glutamic acid and alanine (B). In order to evaluate if the differences in treatment and observed rates of Glu and Ala correlate with apoptosis, a PLS model was generated. Plot (**C**) shows the observed versus predicted values of the model, in which a dataset including Gln, Glu, Ser, Gly, Ala, Cys-Cys and Lys rates was reduced and then correlated with caspase 3 activity [act/h] as apoptotic response. Hence, a significant correlation is shown for caspase 3 activity in untreated (light grey) versus Simvastatin treated (dark grey) A375 cells. Plot (**D**) shows the residuals of the model with a standard deviation of 41.09 [act/h] indicating the good model fit with the observed data.

The correlation between statin treatment and cell growth has already been described in the Figs 1, 2 and 3. The inhibition of the HMG-CoA reductase leads to a significant inhibition of metastatic cell growth which can already be seen after 24 h in A375, 518a2 and 6F cells. The uptake of glutamic acid and alanine not only determines the metabolic differences between the growth velocities, but also the metabolic adaptation after Simvastatin treatment in A375 cells (Fig. 5A,B). Untreated A375 cells take up glutamic acid and alanine. In contrast, the uptake of these two amino acids is not only inhibited in the presence of Simvastatin, it even leads to secretion of alanine and glutamic acid, indicated by a positive value for PC2 (Fig. 5B). The metabolic benefit of glutamic acid uptake by metastatic cells may fuel the energy producing TCA cycle in an anaplerotic reaction^3^. Additionally, alanine functions as a glucogenic amino acid supporting gluconeogenesis. The inhibition of the HMG-CoA reductase by Simvastatin may lead to an accumulation of acetyl-CoA, which is fuelled in the TCA cycle instead of the lipid synthesis. This rewiring of acetyl-CoA could be the reason for the increased secretion of alanine in statin treated A375 cells and may contribute to statin induced apoptosis in particularly fast growing metastatic cells^10,16^. In addition to the amino acid profiles, the activity of caspase 3, 8 and 9 was determined after 24 h and 48 h, to combine the induction of apoptosis with metabolic adaptation (Supplementary Table S1). Via PLS regression, a correlation between specific amino acids rates (Gln, Glu, Ser, Gly, Ala, Cys-Cys and Lys) and caspase 3 activity could be established for the metastatic A375 cells (Fig. 5C,D). Based on the PLS regression the induction of caspase 3 by simvastatin could be predicted and significantly separated from untreated A375 cells (Fig. 5C) and thereby indirectly confirmed previous observations for Simvastatin induced apoptosis^8,10^.

Summing up, the hypothesis is supported that alanine and/or glutamic acid enhance the growth characteristics of melanoma cells.

### Growth effects of glutamic acid and alanine on melanoma cells

**Figure 6:**
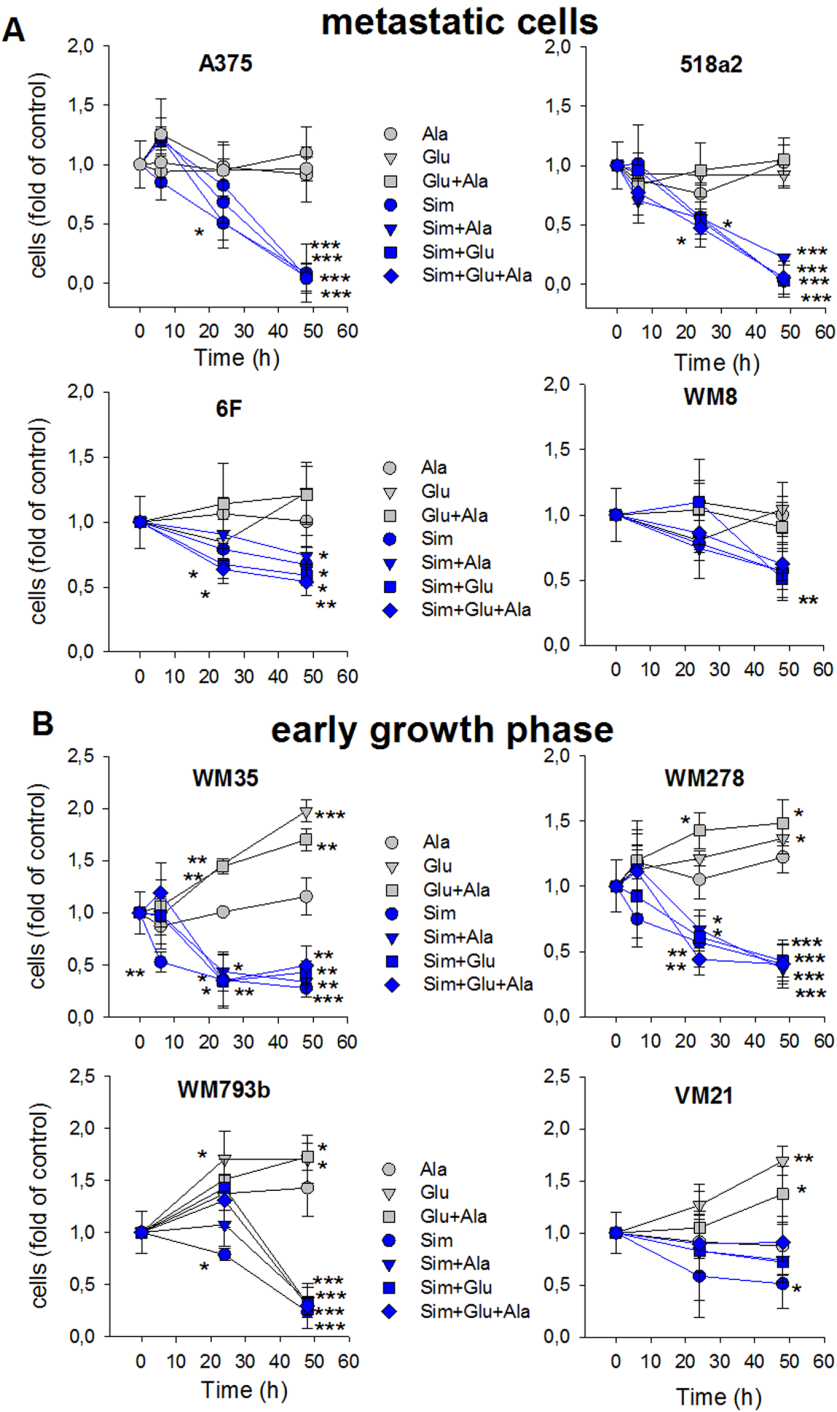
Proliferation of human melanoma cells. Human metastatic melanoma cells (**A**) and melanoma cells from the early growth phase (**B**) were cultured in the absence or presence of L-alanine (100 µg/ml; Ala), L-glutamic acid (200 µg/ml; Glu), L-alanine plus glutamic acid (Ala + Glu) or their combinations with Simvastatin (10 µM; Sim). Data were normalized to the corresponding time points of the untreated controls. Symbols and errors depict the mean ± SD (n = 3). Statistics were performed with one-way ANOVA and post hoc Dunnett’s test. Significance *vs*. control treatment is indicated with asterisks (*p < 0.05, **p < 0.01; ***p < 0.005).

In order to confirm a biological effect of alanine and/or glutamic acid at concentrations determined in conditional media (Supplementary Table S1), the proliferation of slow and fast growing melanoma cell lines was analysed and normalized to proliferation of untreated cells (Fig. 6). Clearly, glutamic acid or the combination of glutamic acid and alanine stimulated proliferation in slow growing WM35, WM278, WM793b and VM21 cells significantly, while alanine *per se* had no or only a weak effect. Conversely, in the fast growing metastatic cells (A375, 518a2, 6F and WM8) the amino acid combinations had no growth stimulatory effect (Fig. 6A). Upon inhibition of the mevalonate pathway by Simvastatin this growth stimulatory effect was completely abrogated in all melanoma cells (Fig. 6). However, in some melanoma cells (A375, 518a2 WM35 and WM793b cells) proliferation is completely inhibited after 48 hours in the presence of simvastatin. Interestingly, under these conditions caspase 3 activation is significantly activated in metastatic cells, while in early growth phase melanoma cells threshold levels were reached, which were significant only for WM278 cells (Supplementary Fig. S2).

**Figure 7:**
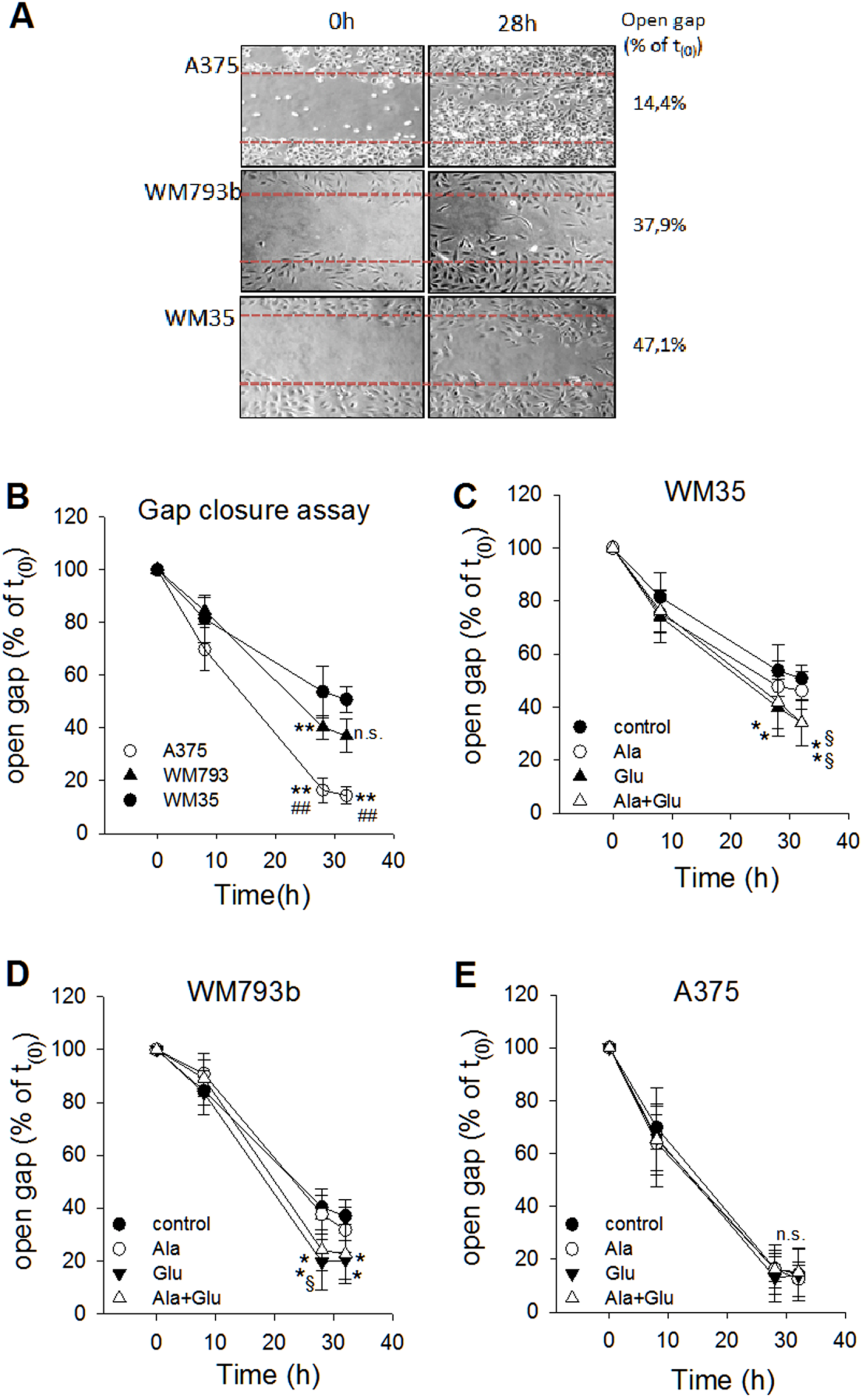
Alanine and glutamic acid enhance migration of slow growing WM35 and WM793b cells. Human melanoma cells, WM35, WM793b and A375, were prepared for scratch assay and then treated in the absence (**A**,**B**) and presence of L-alanine (100 µg/ml; Ala), L-glutamic acid (200 µg/ml; Glu) or their combination (Ala + Glu). Data points represent the mean ± SD (*n* = 4). Statistical significance was performed with one-way ANOVA and post hoc Dunnett’s test; asterisks denote significance versus WM35 cells: *P < 0.05; **P < 0.005, or hatches versus WM793b cells: ^#^*P* < 0.05; ^##^*P* < 0.005 in panel B. In panel C-E asterisks denote significance versus controls: *P < 0.05 or § versus alanine treatment: § *P* < 0.05; n.s. denotes not significant.

**Figure 8:**
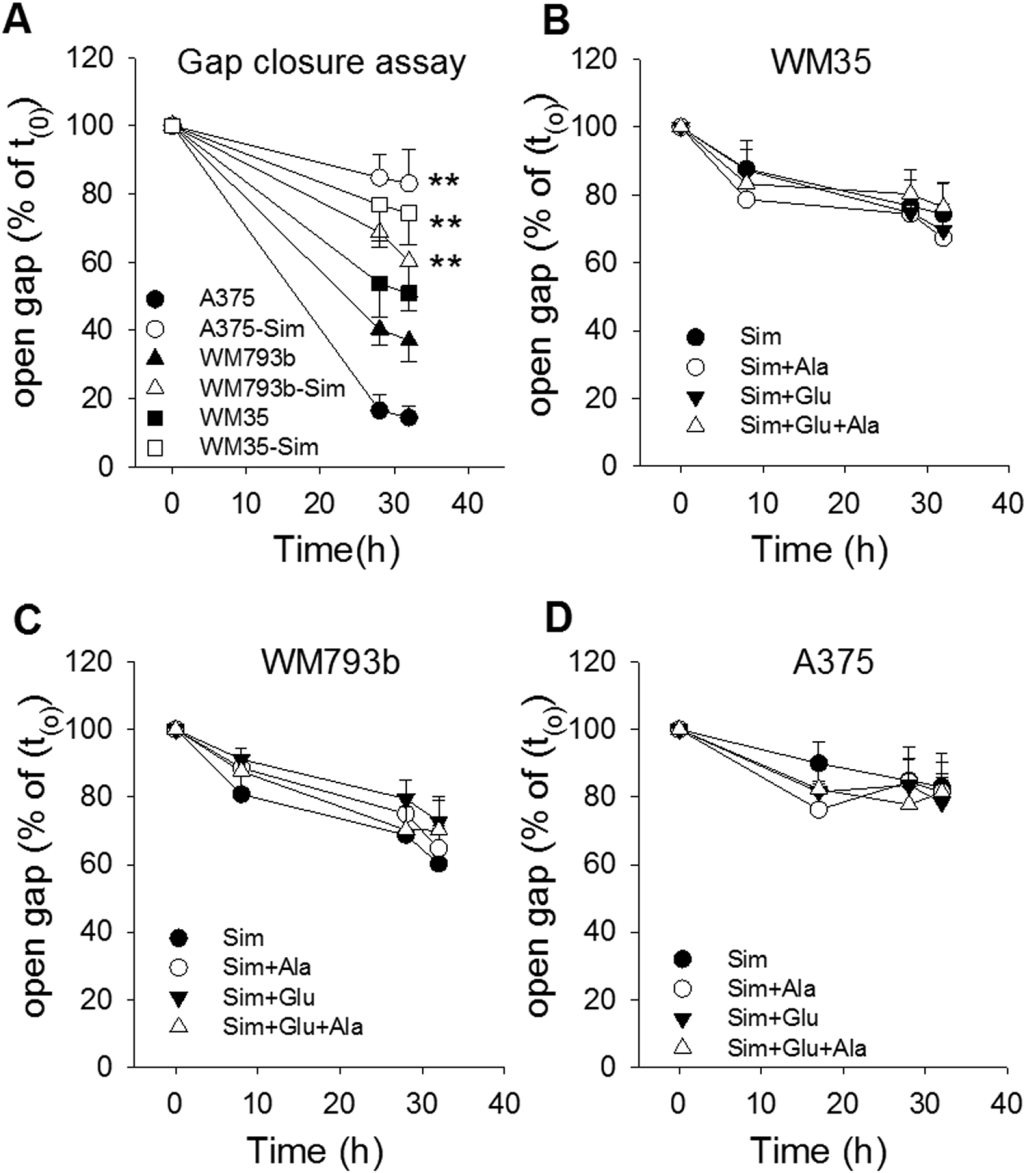
Simvastatin inhibits migration in melanoma cells independent of the growth stage. Human melanoma cells, WM35, WM793b and A375, were prepared for scratch assay and then treated in the absence (**A**) and presence of Simvastatin (10 µM; Sim), L-alanine (100 µg/ml; Ala), L-glutamic acid (200 µg/ml; Glu) or their combinations. Data points represent the mean ± SD (*n* = 4). Statistical significance was performed with one-way ANOVA and post hoc Dunnett’s test; asterisks denote significance versus untreated control cells: *P < 0.05; **P < 0.005.

In order to corroborate growth stimulation of glutamic acid and alanine, migration was investigated in a wound healing assay (Fig. 7). Again, the stage dependent differences in proliferation were mirrored in delayed gap closure of WM35 and WM793b cells (Fig. 7). Gap closure was significantly faster in WM35 and WM793b cells by addition of glutamic acid either in the absence or presence of alanine, while migration of A375 cells was not influenced. Again, Simvastatin significantly inhibited migration of all three melanoma cell lines (Fig. 8A). However, the effect was less pronounced in WM35 and WM793b cells. The addition of glutamic acid, alanine or a combination was not effective in accelerating migration (Fig. 8B–D).

**Figure 9:**
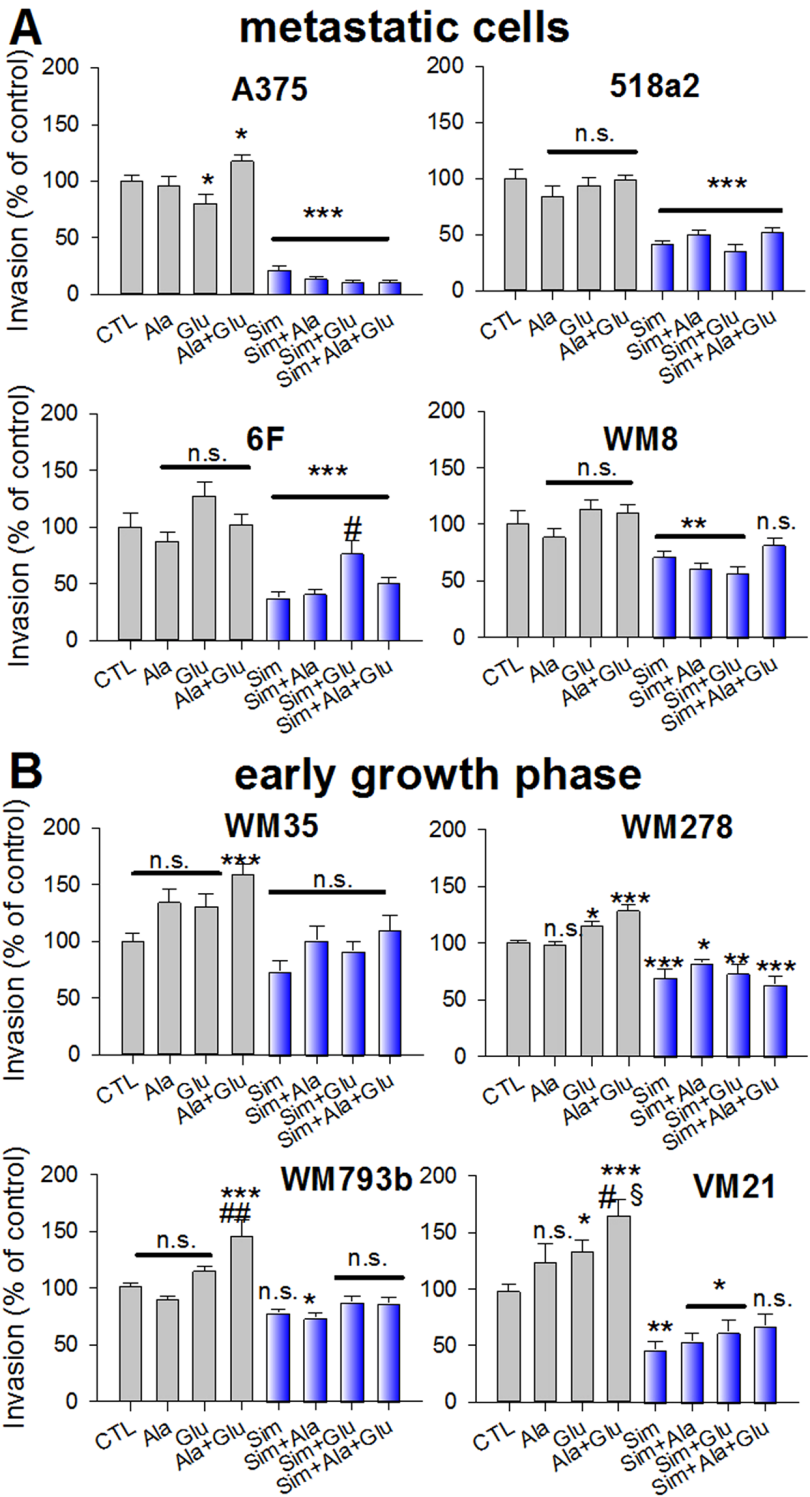
Alanine and glutamic acid significantly stimulate invasion in slow growing (**B**), but not in metastatic melanoma cells (**A**). Human melanoma cells (3,5*10^4^/well) were kept in collagen I coated inner Boyden chambers for 24 hours. The medium in the outer chamber (CTL) was supplemented by L-alanine (100 µg/ml; Ala), L-glutamic acid (200 µg/ml; Glu), L-alanine plus glutamic acid (Ala + Glu) or their combinations with Simvastatin (10 µM; Sim). Data were adjusted for proliferation and normalized to the corresponding time points of the untreated controls. Bars and errors depict the mean ± SEM (n = 4). Significance *vs*. control treatment was performed with one-way ANOVA and *post hoc* Student-Newman-Keuls test and indicated with asterisks (*p < 0.05, **p < 0.01, ***p < 0.005). Significance *vs*. L-alanine or L-glutamic acid were indicated with # or §, respectively (^#^p < 0.05, ^##^p < 0.01, ^§^p < 0.05; n.s. denotes not significant).

Finally, we investigated the invasion of melanoma cells to expand amino acid effects on aggressiveness and malignancy. Within 24 hours invasion of slow growing melanoma cells (WM35, WM278, WM793b and VM21) was significantly enhanced in the combination of glutamic acid and alanine (Fig. 9B). In contrast, invasion of fast growing metastatic melanoma cells was not altered by glutamic acid and alanine addition. Although invasion of A375 cells was significantly enhanced by the combination of glutamic acid and alanine, these data are considered inconclusive, since glutamic acid alone significantly reduced invasiveness (Fig. 9A). Again, inhibition of the mevalonate pathway significantly inhibited invasion of metastatic melanoma cells, while in slow growing melanoma cells this was only seen in WM278 cells (Fig. 9).

Taken together, these data confirm that an inhibition of the mevalonate pathway arrests proliferation, migration and invasion of melanoma cells, which is not compensated by amino acid rescue metabolism like glutaminolysis.

## Discussion

In the present study we have demonstrated that: (i) amino acid profiles from conditional media of melanoma cells from different growth stages are significantly different in their composition; (ii) the differences are of biological significance and corroborate acceleration of proliferation, migration and invasion by glutamic acid in slow growing melanoma cells, but not in metastatic cells; and (iii) inhibition of the mevalonate pathway overcomes the growth stimulatory effect of glutamic acid and alanine, highlighting the stringent dependency on this pathway in melanoma cells.

A central issue of transformed cells, including melanoma, involves high glycolytic flux, which transforms glucose to lactate^3^. Stimulation of this pathway branches also into the pentose phosphate pathway and the serine synthesis pathway^24^. The latter is of particular interest in melanoma because the first reaction in this pathway is catalysed by phosphoglycerate dehydrogenase (PHGDH), which is amplified in melanoma^25^. Of note, a decrease in PHGDH expression reduced proliferation not only in melanoma cells but also in breast cancer cells, and heterologous overexpression in non-transformed cells predisposes cells to transformation^24,25^. Although these studies link a glycolytic enzyme to proliferation of cancer cells, serine or glycine were not identified as discriminators for differences in growth behaviour in the melanoma cells investigated in our study.

Under hypoxic conditions tumour cells accumulate lactate, the so called Pasteur effect^26^. Although we have not determined lactate, we could measure high levels of acetate in the conditional media. It has been previously shown that acetate is the main contributor to the formation of acetyl-CoA to fuel fatty acid synthesis^27^. However, lipid synthesis starting from acetyl-CoA branches also into the mevalonate pathway, which is responsible for the synthesis of integral intermediates for proper tumour growth and progression^7^. We have previously shown that statins reduce the dolichol levels in human neuroblastoma cells and thereby reduce the glycosylation of integral membrane proteins like the ATP-binding cassette transporter (ABCB1; P-glycoprotein)^11,15^. Another crucial link between tumorgenesis and glucose consumption has been elucidated by Cheng *et al*., showing that increased glucose uptake leads to N-glycosylation of the sterol regulatory element-binding protein (SREBP) cleavage-activating protein (SCAP) and consequently in lipogenesis via activation of SREBP-1^28^. Indirectly, our results confirm the crucial role of the mevalonate pathway in proliferation, migration and invasion (Figs 1, 6, 8 and 9). Simvastatin significantly inhibited the proliferation and migration of melanoma cells, which was not compensated by the co-application of glutamic acid and alanine. Interestingly, invasion was also significantly suppressed by Simvastatin in all cell lines, except WM35 cells (Fig. 9). Blockade of invasion might be explained by inhibition and reduction of matrix metalloproteinases in non-transformed endothelial cells and cancer cells including melanoma cells^29–31^. Although epidemiological analyses have revealed a beneficial effect of statins in human cancers including melanoma, opposite reports exist^7,32,33^. Importantly, statins have been discussed as potential candidates for chemoprevention, alone or in combination with other drugs, like metformin or cyclooxygenase inhibitors^7,12^. Nevertheless, statins are well tolerated but were evaluated for cholesterol lowering in cardiovascular risk patients, thus other derivates may exist with improved anticancer action.

Mitochondrial glutaminolysis overcomes the decrease of TCA cycle metabolites under hypoxic conditions^34^. The glutamine uptake and consecutive activation of the isocitrate dehydrogenase 1 provides sufficient acetyl-CoA to support lipogenesis^3,6^. In this study, we have identified glutamic acid together with alanine as the primary determinates for discrimination between melanoma cells from different growth stages in a multivariate analysis. Glutamic acid is known to be taken up by non-transformed cells and tumor cells^35^. However, a specific function or an extracellular signaling cascade are not attributed to this amino acid. Interestingly, glutamic acid enhanced proliferation, migration and invasion are only observed in slow growing melanoma cell lines, but not in metastatic melanoma cells (Figs 6–9). An explanation of this phenomenon is currently not available. Nevertheless, dualistic effects have been described for the transcriptional coactivator peroxisome proliferator-activated receptor-gamma coactivator 1 α (PGC1α), the master regulator of mitochondrial biogenesis^36^. High levels of PGC1α, found in the radial growth phase of melanoma were assigned to promote growth and survival, while low levels were associated with invasion and metastasis. The authors conclude that signaling inputs including nutrients may guide changes in PGC1α and thereby switch from survival and proliferation to invasion and metastasis^36^.

Alanine is considered a glycogenic amino acid, but a possible role for secreted alanine is currently not available. In our experiments, the biological effects of alanine alone were not significant in any melanoma cell line tested, indicating that the glycogenic effect is of minor importance to proliferation and migration (Figs 6 and 7). Nevertheless, secretion of alanine has been previously shown for melanoma cells but not for melanocytes^5^. Besides, we could identify an enhanced uptake of proline by slow growing, non-metastatic melanoma cells (Fig. 4). A source for extracellular proline *in vivo* is provided by its abundance in collagen, which is part of the extracellular matrix and therefore crucial for the tumour microenvironment^23^. Conversely, proline may be used as a source for ATP synthesis by collagen breakdown during tumour cachexia. Nevertheless, intracellular proline biosynthesis has been previously described in mitochondrial glutaminolysis or alternatively from cytosolic ornithine in WM35 and WM 793 cells^22^.

Here we used multivariate data analysis as a tool, suitable to identify crucial amino acids from media of melanoma cell lines of different stages with specific biological characteristics. Our results confirm for the first time that amino acid profiles from conditional media are capable to distinguish between stage dependent growth and treatment. Hence, changes in amino acid composition of media from melanoma cells derived from different disease states reflect a stage dependent adaptation with functional consequences on proliferation, migration, aggressiveness and survival. Thus, identification of glutamic acid mediated signalling may open pharmacological targeting in early melanoma stages.

## Materials and Methods

### Materials

Simvastatin was purchased from Merck^®^ (Rahway, N.J., USA), and the fluorescent substrates for caspases 3, 8 and 9 were from Alexis Biochemicals^®^ (San Diego, CA, USA). All other chemicals were of analytical grade and obtained from Sigma Chemical Co.^®^ (St. Louis, MO, USA).

### Cell culture

Human melanoma cell lines A375, 518a2, 6F (a generous gift by Dr. Christoph Hoeller, Department of Dermatology, Medical University Vienna) and WM8 were kept in Dulbecco’s modified Eagle medium-high glucose (DMEM; Sigma Chemical Co.^®^, St. Louis, MO, USA) supplemented with 1% penicillin/streptomycin and 10% fetal calf serum. Human melanoma cell lines WM35, WM278 and WM793b were kept in a 1:1 mixture of DMEM -high glucose and Ham’s F12 medium (Sigma Chemical Co.^®^, St. Louis, MO, USA) supplemented with 1% penicillin/streptomycin and 2% fetal calf serum. The VM21 cell line (a generous gift from Dr. Robert Eferl and Dr. Walter Berger, Cancer Research Institute, Department of Applied and Experimental Oncology, Medical University Vienna) was kept in RPMI-1640 medium (Sigma Chemical Co.^®^, St. Louis, MO, USA) supplemented with 1% penicillin/streptomycin and 10% fetal calf serum and^37^. Cells were maintained at 37 °C in a 5% CO_2_ humidified atmosphere. For HPLC-analyses of conditional media all cells were kept in DMEM-high glucose (Sigma Chemical Co.^®^ (St. Louis, MO, USA)) supplemented with 1% penicillin/ streptomycin and 10% fetal calf serum.

### Cell Proliferation

Melanoma cells were seeded in a 24-well plate at a density of 3.5 × 10^4^ cells per well. After an overnight recovery, cells were incubated with indicated compounds. The number of attached (living) cells was determined in duplicates at given time points. Attached cells were washed with PBS, detached with trypsin and resuspended in medium (500 µl) for automated cell counting (Luna II cell counter, Logos Biosystem, Villeneuve-d’Ascq, France). Data from three independent experiments were pooled. As a comparison, proliferation was also determined in DMEM-high glucose medium for all cell lines described above (data not shown).

### Scratch assay

Melanoma cells were seeded in a 12-well plate at a density of 7 × 10^5^ cells per well. After an overnight recovery, the cell monolayer (70–80% confluence) was scratched off with a plastic pipette tip (200 µl) on the bottom of each well. Cells were washed twice with PBS and treated with compounds indicated in the figure legends. At the indicated points of time four pictures were taken from each well at labelled orientation points and three wells were measured for each condition. Three independent experiments were carried out. The percentage of cell-free surface was calculated by the TScratch software tool (CSElab, ETH Zürich, Switzerland) and normalized to the gap at the beginning of the experiment (t_(0)_).

### Invasion assay

Membranes of Boyden chambers were coated with type I collagen (Thermo Scientific®, Waltham, MA, USA; 300 µg/ml in PBS/NaOH) and kept overnight at 37° under aseptic conditions. Covered membranes were washed with PBS and 3,5*10^4^ cells per well were allowed to adhere for 6 hours. Thereafter, the indicated compounds were added to the outer chamber at 37 °C in a 5% CO_2_ humidified atmosphere for 24 hours. Thereafter, cells from the inner membrane were removed and invaded cells from the outer membrane were fixed (4% PFA/PBS), washed (PBS) and stained with Hoechst 33342 dye (20 µM). Membranes were mounted on a glass cover slip and 5 images were taken from one membrane using a fluorescence microscope Axiovert 200 M (Zeiss®; Oberkochen, Germany) at a magnification of 100x. Cell counting was performed with ImageJ software (www.imagej.net) and corrected for the individual proliferation rates of the cells. Data were normalized to the invasion of cells under control conditions.

### Quantification of amino acids and metabolites

Quantification of substances in the cell-free microenvironment was performed via HPLC analysis using an Ultimate 3000 (Thermo Fisher Scientific, USA) system equipped with a pump (LPG-3400SD), a split-loop autosampler (WPS-3000 SplitLoop), a column oven (Col.Comp. TCC-3000SD) and a fluorescence detector (FLD-3400RS). Chromeleon 7.2 was used for the control of the device as well as for the quantification of the peak areas.

Chromatographic separation of amino acids was achieved with a reversed phase column (Agilent Eclipse AAA, 3 × 150 mm, 3.5 µm), a guard column (Agilent Eclipse AAA, 4.6 × 12.5 mm, 5 µm) and a gradient using eluent (A) 40 mM NaH_2_PO_4_ monohydrate pH 7.8 and eluent (B) MeOH/ ACN/ H_2_O (45/45/10, v/v/v)^38^. The protocol was run with a flowrate of 1.2 mL min^−1^, the column oven temperature was set to 40 °C and the injection volume was 10 µL. As most amino acids have no fluorophore in their structure an in-needle derivatization step was performed using 0.4 M borate buffer, 5 mg mL^−1^ ortho-phthaldialdehyde (OPA) in 0.4 M borate buffer containing 1% of 3-mercaptopropionic acid (3-MPA), 2.5 mg mL^−1^ fluorenylmethyloxylcarbonyl chloride (FMOC) and 1 M acetic acid for pH adjustment. In order to guarantee sample quantification despite the derivatization step, every sample and standard were spiked with 25 mM sarcosine and 25 mM norvaline both in 0.1 M HCl as internal standards. Primary amines and norvaline were detected at Ex 340 nm/Em 450 nm and secondary amines and sarcosine were detected at Ex 266 nm/Em 305 nm. Additionally, quantification and calibration were performed with an external standard including 22 amino acids.

Acetate and Pyruvate were separated with an Aminex HPX-87H (Bio-Rad) column and guard column Aminex HPX 87-H. The method was run isocratically with 0.1% TFA in H_2_O at 50 °C for 60 minutes with a flowrate of 0.6 mL min^−1^. Organic acids were detected at 210 nm and quantified by external calibration. All chemicals used for HPLC were of highest quality or HPLC grade quality. For the chromatographic method ultra-pure water was used, produced with a Milli-Q system from Merck Millipore (Billerica, USA).

### Caspase activity measurements

Caspase activity was measured with specific fluorescent caspase 3, 8 and 9 substrates, as previously described^9,10,15^.

### Calculation of specific rates

Specific rates for amino acids and organic acids were calculated from 24 h and 48 h values (Supplement Table S1), in order to allow the comparison of the data. The following formula was used:

$${q}_{i}=\frac{1}{X}\cdot \frac{dci}{dt}$$

*X* is representing the cell count, *dc*_*i*_*/dt* is the change of the concentration of substance i over time and *q*_*i*_ is the specific rate of substance i. A positive specific rate of substance i shows a secretion of i by the cells, whereas a negative rate represents an uptake of i by the cells (e.g. see *PC4 in* Fig. 4B*; proline uptake by WM793b cells*.).

### Statistical analyses

The experiments were performed at least three times, carried out at least in duplicates and presented as mean ± standard deviation, if not otherwise stated. Statistical analysis for multiple comparisons was done by one-way ANOVA, followed by *post hoc* Tukey’s, Dunnett’s or Student-Newman-Keul´s test (GraphPad Prism^®^ software). Pairwise comparison was done with Mann-Whitney Rank Sum Test or Students T-test. A value of p < 0.05 was considered as statistically significant.

Multivariate data analysis was performed using Umetrics SIMCA 4.0 software (Umeå, Sweden). Data were normalized and mean-centred before principle component analysis (PCA) or partial least squares (PLS) regression. Models were generated for all data points or after rate calculation between 24 h and 48 h of incubation. PCA and PLS were used as statistical methods for the deconvolution of correlations in big datasets. The here presented dataset (Supplementary Table S1) includes more than 20 variables (amino acids and organic acids). In order to evaluate the linkage between these variables and the observations (i.e. the cell lines with and without Simvastatin treatment at different time points) statistically, the data set was restructured along the highest variance in the data. This new structure is represented by the generated principal components (PC) for PCA or latent variables (LV) for PLS. Interpretation of the new data structure is possible with the score plot, showing the distribution of the observations between two PCs. As example, we get 4 PCs, where each PC represents another variance in the dataset (e.g. differences in the uptake and secretion of amino acids). The highest variance is represented by PC 1, then PC 2, etc. If a score plot of PC 1 versus PC 2 is created the observations (e.g. cell lines with or without treatment) are distributed in this new coordinate system according to their differences that are illustrated by PC 1 and PC 2 (e.g. Supplementary Fig. S1). Depending on these differences clustering of the observations in the score plot can be observed. In order to understand which PC represents which variance, the loading plot has to be evaluated. The loading plot shows the distribution of the variables (e.g. amino acid rates) in the new coordinate system, hence, between PCs and gives information about the variables responsible for variance/clustering of the observations (e.g. Fig. 3D)^39^. A high value for a variable in the loading plot for PC 1 means that observations that cluster along PC 1 show a high rate/concentration of this variable. Hence, hypotheses about correlations between metabolic behaviour and cell lines and their treatment can be generated.

The main difference between PCA and PLS is that PCA just reduces a dataset (e.g. amino acid rates of different cell lines) whereas PLS additionally allows correlations of this dataset with a response (e.g. caspase activity; Fig. 5). Hence, we can assume which variable (amino acid) correlates with higher or lower caspase activity in certain cell lines by the so-called coefficient plot. The model created via the PLS algorithm is then evaluated by plotting the observed values against the predicted values by the model (Fig. 5C). The residuals show how much the observed values vary from the model (Fig. 5D). Both plots give good estimation of the model quality.

## Electronic supplementary material

Supplementary Data
