# Supplementary material for: Amino Acid Signature in Human Melanoma Cell Lines from Different Disease Stages
Source: Sci Rep. 2018 Apr 19;8:6245. doi: 10.1038/s41598-018-24709-0 (PMC5908844; doi:10.1038/s41598-018-24709-0)
Supplement: Supplementary file 1 — Supplementary Data [file 41598_2018_24709_MOESM1_ESM.pdf]

AMINO ACID SIGNATURE IN HUMAN MELANOMA CELL LINES FROM DIFFERENT DISEASE STAGES

Wasinger Christine[1+]*, Hofer Alexandra*[2+]*, Spadiut Oliver*[2] *and Hohenegger Martin*[1*]

[1]Institute of Pharmacology, Center for Physiology and Pharmacology, Medical University Vienna, Waehringerstrasse 13A, A-1090 Vienna, Austria

[2]Institute for Chemical, Enviromental and Biological Engineering, TU Wien, Gumpendorferstrasse 1a, A-1060 Wien, Austria

[+]These authors contributed equally to the work.

*Correspondence should be addressed to M.H. (email: martin.hohenegger@meduniwien.ac.at)

Supplementary Table S1: Amino acids, metabolites and caspase activity used for multivariate analyses.

		Pyruvate		Acetae		Glutamine		Asparagine		Serine		Glycine		Threonine		Tyrosine		Cystine		Caspase 3		Caspase 8		Caspase 9	
		µg/l				µg/l														OD./µg/min					
A375		mean	SD	mean	SD	mean	SD	mean	SD	mean	SD	mean	SD	mean	SD	mean	SD	mean	SD	mean	SD	mean	SD	mean	SD
24h	CTL	68,5	12,9	908,4	179,5	1898,8	266,1	22,2	4,2	78,1	12,8	68,1	11,5	190,6	32,7	139,5	24,5	77,9	14,8	903,4	71,8	368,5	18,6	1640,4	664,4
24h	10µM Sim	107,4	28,5	1305,9	336,8	3103,9	697,4	41,3	9,8	141,3	34,3	102,2	24,2	307,3	74,5	224,9	54,9	127,9	34,3	3528,1	411,5	355,4	60,2	2386,7	468,3
48h	CTL	39,2	7,7	316,4	62,1	711,0	127,6	8,5	1,6	15,9	2,7	21,8	3,9	74,8	13,6	53,3	9,7	29,3	6,9	1292,3	227,4	333,6	24,5	1641,4	547,3
48h	10µM Sim	79,9	19,2	1124,1	324,9	2356,9	567,6	39,8	9,6	112,5	27,5	89,1	21,4	252,8	61,9	182,0	44,5	96,0	24,9	17214,4	1990,8	1549,1	178,2	4417,0	1041,5
WM793																									
24h	CTL	591,7	64,6	591,4	66,5	6698,3	604,9	67,0	11,6	277,3	48,2	238,0	41,2	665,4	106,5	489,3	79,2	250,3	21,2	1605,2	694,3	1582,0	709,1	1121,6	132,6
24h	10µM Sim	629,0	116,5	652,0	117,5	7782,0	1389,9	81,4	14,5	336,1	63,1	291,4	58,3	777,5	140,9	563,0	105,6	291,0	65,9	1878,1	384,6	1919,2	886,9	1097,8	357,2
48h	CTL	391,9	83,3	364,8	89,1	2832,5	659,1	31,6	5,8	112,9	27,2	123,7	31,3	315,8	59,9	229,5	43,8	132,0	31,3	1311,8	536,1	1349,8	665,1	767,5	185,2
48h	10µM Sim	689,4	164,1	774,3	173,7	7668,5	2056,4	91,6	23,3	337,6	81,4	329,0	77,3	792,1	193,9	571,7	143,1	308,3	69,9	4199,0	2509,2	2383,9	1754,7	2713,7	2132,9
WM35																									
24h	CTL	431,0	62,1	761,5	108,3	4940,8	800,9	49,4	7,7	238,2	37,4	180,5	29,3	549,5	90,1	406,0	64,7	253,6	56,8	937,2	267,2	1587,4	408,1	766,4	234,2
24h	10µM Sim	490,2	95,7	866,0	143,2	6298,5	1721,4	57,7	13,4	277,2	54,3	217,4	41,2	633,7	142,3	461,0	101,2	234,0	49,2	975,1	269,0	1766,5	445,5	701,1	167,0
48h	CTL	419,7	173,4	502,9	146,0	2793,0	792,7	24,1	8,5	119,0	38,9	98,7	32,2	312,5	101,7	228,5	75,3	120,6	38,4	1189,9	405,2	1750,4	189,3	874,7	58,8
48h	10µM Sim	532,0	140,1	879,3	283,3	5783,6	1707,4	56,8	15,2	238,7	68,0	208,4	55,9	604,8	179,7	437,4	128,6	231,0	78,6	1209,4	324,4	1875,7	342,1	951,5	145,8

		Glutamic acid		Aspartic acid		Histidine		Arginine		Lysine		Alanine		Valine		Methionine		Phenylalanine		Isoleucine		Leucine		Tryptophan		Proline	
		µg/l		µg/l		µg/l						µg/l															
A375		mean	SD	mean	SD	mean	SD	mean	SD	mean	SD	mean	SD	mean	SD	mean	SD	mean	SD	mean	SD	mean	SD	mean	SD	mean	SD
24h	CTL	106,0	18,6	13,4	2,6	87,7	15,4	428,8	73,2	240,6	41,8	71,3	12,3	146,3	24,7	54,6	10,3	123,2	20,8	153,8	26,6	168,4	28,3	22,4	4,4	71,0	12,5
24h	10µM Sim	155,8	37,9	20,9	5,3	139,7	33,8	675,2	164,5	379,7	89,0	104,7	24,0	243,3	60,0	92,0	25,0	202,3	48,5	257,6	62,9	283,9	69,4	34,9	8,8	118,1	30,1
48h	CTL	51,0	9,3	5,9	1,2	33,7	6,1	166,7	31,3	87,8	16,1	54,4	9,9	49,3	9,5	16,1	4,1	45,4	8,4	56,3	10,1	56,2	10,1	9,8	2,0	43,0	7,4
48h	10µM Sim	202,9	49,1	17,1	4,1	115,3	28,2	540,1	131,1	304,9	83,0	135,2	31,9	193,7	48,1	75,7	18,8	164,0	40,8	205,2	51,4	227,9	55,6	28,5	6,9	111,3	28,2
WM793																											
24h	CTL	439,4	72,2	44,4	4,8	302,7	46,7	1486,0	244,5	812,3	112,9	72,4	13,3	571,9	90,6	199,5	22,3	445,0	73,9	653,1	104,0	703,8	116,0	74,0	8,1	221,7	37,4
24h	10µM Sim	489,1	89,9	51,9	9,5	359,6	65,9	1711,2	311,4	952,3	177,5	98,0	22,2	662,9	121,1	239,4	44,9	511,5	92,0	755,5	137,1	816,7	149,2	86,6	15,9	260,5	48,0
48h	CTL	275,0	48,4	22,4	5,8	147,8	27,4	699,3	134,4	369,7	123,2	74,7	18,2	264,1	49,8	93,2	19,8	201,8	40,2	296,7	57,8	316,2	63,7	37,4	9,6	104,6	17,0
48h	10µM Sim	749,6	175,8	51,8	12,5	374,6	91,0	1718,3	416,5	1051,6	262,3	162,9	37,0	676,4	161,1	250,9	51,6	508,5	121,4	750,5	179,7	829,9	198,1	86,3	20,8	244,7	78,3
WM35																											
24h	CTL	320,7	51,0	36,3	5,4	253,3	42,1	1226,4	199,3	682,0	116,1	58,1	9,1	486,0	80,1	181,7	28,8	363,4	58,6	530,3	88,3	572,5	94,8	60,5	9,0	214,2	36,3
24h	10µM Sim	350,8	83,7	42,0	8,2	293,0	66,1	1421,0	313,7	773,5	160,2	72,9	13,2	540,4	121,7	198,5	46,6	420,0	93,2	609,2	132,2	657,6	143,8	70,0	13,7	233,6	53,1
48h	CTL	257,4	89,2	24,1	9,3	144,0	48,5	716,3	233,7	382,3	130,4	75,2	32,4	252,7	80,0	95,7	32,8	202,9	65,2	284,8	92,8	302,6	97,7	40,2	15,5	154,5	49,6
48h	10µM Sim	497,2	136,0	40,5	12,2	279,8	81,9	1352,3	384,2	705,0	203,9	153,5	41,3	500,5	146,1	177,2	49,3	392,1	114,8	553,8	163,1	597,5	172,1	67,5	20,3	283,4	86,4

Amino acids and metabolites were determined in the conditional media from human melanoma cells (WM35, WM793 and A375) in the absence and presence of Simvastatin (Sim; 10 µM) after 24 and 48 hours of incubation. Caspase 3, 8 and 9 activities were determined under identical conditions and time points in WM35, WM793 and A375 cells. Data represent the mean and SD of 4-6 experiments and were used for multivariate analyses.

Supplementary Figure S1: In detail analysis of the score plot of human melanoma cell lines derived from Fig. 3A.

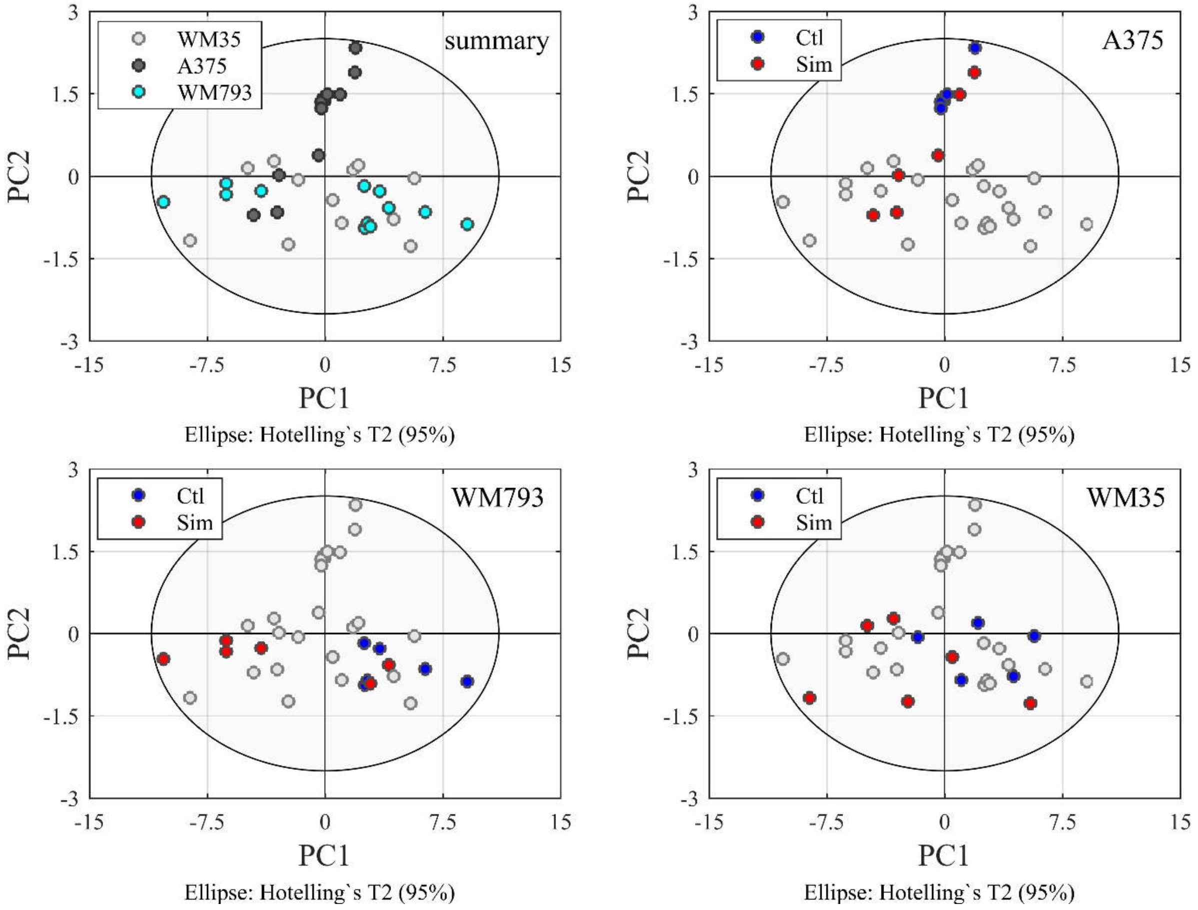

In this score plots human melanoma cell lines were depicted according to PC1 and PC2 from Fig. 3A. Colour coding delineates the contribution of each cell line (summary: all cell lines; A375, WM793 and WM35) in the absence (CTL) and presence of 10 µM Simvastatin (Sim).

Supplementary Figure S2: Simvastatin induced caspase 3 activity in primary and metastatic human melanoma cells.

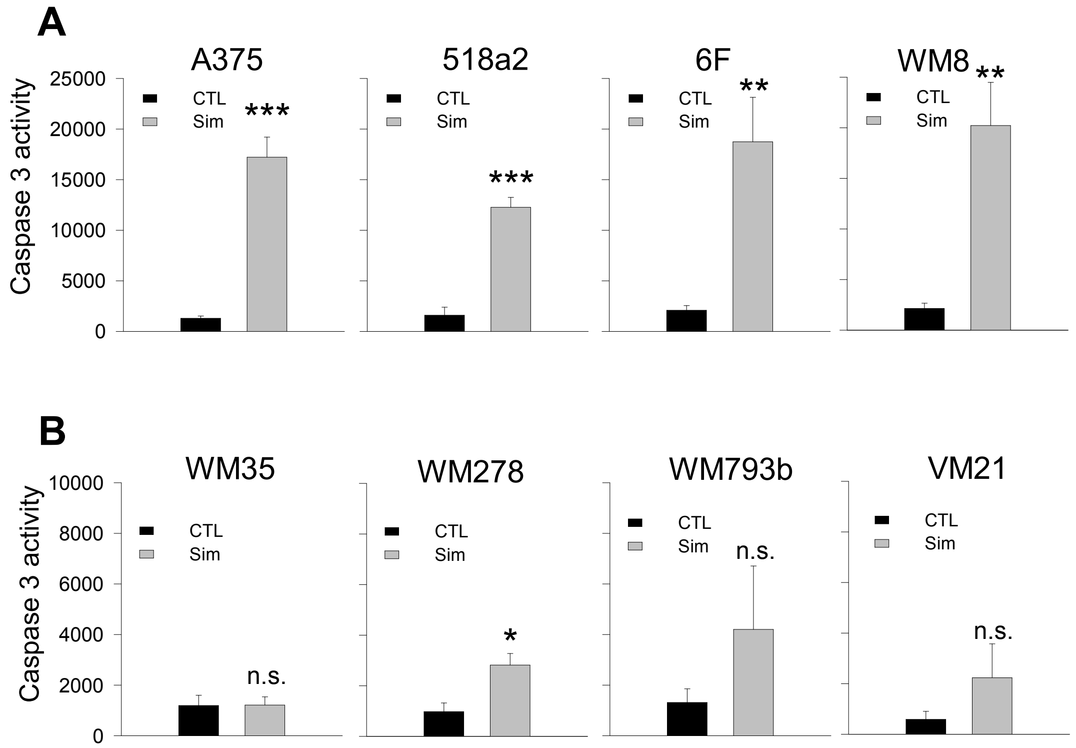

Metastatic (A) and primary human (B) melanoma cells were treated in the absence (CTL) and presence of 10 µM Simvastatin (Sim) for 48 hours. Cells were lysed and analysed for caspase 3 activity (OD./µg/min) according to references in the materials and methods section using a cleavable fluorescent substrate specific for caspase 3. Assays were performed in triplicates. Bars and errors represents the mean ± S.D. (n=5). Statistical significance was performed with Students T-test (n.s.: not significant; *: $p<0.05$; **: $p<0.005$; ***: $p<0.001$).
